# Toward Socially Meaningful Case Conceptualization: The Risk-Driven Approach

**DOI:** 10.1007/s40617-023-00812-1

**Published:** 2023-05-24

**Authors:** Rachel S. Taylor, Richard A. Colombo, Michele Wallace, Benjamin Heimann, Ashton Benedickt, Allyson Moore

**Affiliations:** Center for Applied Behavior Analysis, 11150 West Olympic Blvd., Los Angeles, CA 90064 USA

**Keywords:** Risk, Applied behavior analysis, Case conceptualization, Ethics, Outcomes, Social validity

## Abstract

The Behavior Analysis Certification Board (BACB) *Ethics Code* states that “behavior analysts should put compliance with the law and clients’ interests first by actively working to *maximize desired outcomes and minimize risk*” (emphasis added; BACB, 2020, p. 5). In turn, board certified practitioners must approach the case conceptualization process in applied behavior analysis (ABA) with respect to minimizing risks to an improved quality of life (QoL). As such, ABA services must be based on an understanding of *risk*—risk to ensuring *desired outcomes*. The purpose of the current article is two-fold (1) revisit social validity and propose features of socially meaningful case conceptualization, and (2) introduce a corresponding structured risk-driven approach to ABA service delivery. A primary aim is to equip all stakeholders with readily accessible practice-related supports—ensuring clients’ rights to effective services towards an improved QoL.

The Behavior Analysis Certification Board (BACB) *Ethics Code for Behavior Analysts* states that in “all instances of interpreting and applying the Code, behavior analysts should put compliance with the law and clients’ interests first by actively working to *maximize desired outcomes and minimize risk*” (emphasis added; BACB, 2020, p. 5). As such, board certified practitioners must approach the case conceptualization process in applied behavior analysis (ABA) based on an understanding of *risk*—risk to ensuring *desired outcomes*. Doing so necessitates clearly defined best practice standards for determining appropriate individualized ABA service *goals* as well as the related potential risks towards an improved quality of life (QoL).^1^ Without understanding the environmental variables influencing risk to maximizing desired *outcomes*, ABA services may fail to result in socially meaningful improvements. This undertaking necessitates addressing a few topics that may need further support in the field.

One concern facing the field is the lack of a shared understanding for the defining features of *socially meaningful case conceptualization* (SMCC). This is of particular concern for newer practitioners (certified in the last 5 years) who are expected to make appropriate ABA service recommendations with potentially limited guidance. Previous authors have addressed these and related concerns by focusing on topics such as social validity (e.g., Wolf, 1978) and effective treatment (e.g., Van Houten et al., 1988). Recently enhanced practice standards and increased regulatory requirements also provide further related professional practice supports (e.g., Behavioral Health Center of Excellence [BHCOE], 2022; the Autism Commission on Quality, 2022; *Council of Autism Service Providers* [CASP], 2020). Still, there remains no practitioner resources, known to the current authors, specifically designed to meet the BACBs ethical charge to be actively working to *maximize desired outcomes and minimize risk*—Establishing a mutually agreed upon shared understanding for all aspects of the SMCC process is the first necessary step to this end.

Another concern facing the field of ABA is the rapidly increasing number of board certified behavior analysts (BCBA). Although more recent graduates of master’s degree programs in ABA may be more likely to have read recent literature, many challenges remain for newer supervisors. Fifty percent of current BCBAs have been certified within the last 5 years (BACB, n.d.), setting the occasion for many clients to experience the case conceptualization process under the supervision of someone who is a relatively new practitioner with potentially limited support from more experienced professionals (e.g., Reed & Henley, 2015; Colombo et al., 2020a, b). This current professional practice landscape is especially concerning when contrasted with other human service disciplines. For example, consider a team of medical doctors and nurse practitioners working in a hospital setting where everyone was licensed within the past few years with limited to no access to on-going supervision from more senior level practitioners/mentors (e.g., a chief medical officer). Increasing demand for ABA services under the conditions of limited available expertise may set the occasion for a wide range of novel challenges related to protecting the integrity of ABA^2^ and in turn, ensuring a client's rights to effective treatment defined as follows:

. . . individuals who are recipients or potential recipients of treatment designed to change their behavior have the right to: (1) a therapeutic environment, (2) services whose overriding goal is personal welfare, (3) treatment by a competent behavior analyst, (4) programs that teach functional skills, (5) behavioral assessment and ongoing evaluation, and (6) the most effective treatment procedures available. (Van Houten et al., 1988, p. 381)

Deochand et al. (2020) articulated some concerns with the growing number of BCBAs and corresponding need for more readily accessible practitioner supports and resources. Recent related publications encourage reliance on structured approaches such as risk-benefit analyses (Deochand et al., 2020; Clancy & Plavnick, 2020; Hajiaghamohseni et al., 2022; Plavnick et al., 2020), decision models (Axelrod et al., 1993; Colombo et al., 2020a, b; Cox & Brodhead, 2021; Cox et al., 2020), and other easy-to-use reference materials (Hanley, 2012; Rodriguez, 2020; Vanselow & Bourret, 2012) to name a few. Although these and similar publications offer practical strategies to help practitioners maintain ethical compliance,^3^ the BACBs initial introductory statement regarding risk does not focus on *procedural* risk alone. Instead, behavior analysts are ethically obligated to be “. . . actively working to *maximize desired outcomes* and *minimize risk*” (emphasis added; BACB, 2020, p. 5). This statement is referencing *risk to desired ABA service outcomes*, not just the risk(s) associated with conducting specific procedures (e.g., an experimental functional analysis, or a particular teaching/prompting strategy). As such, a practitioners’ obligations regarding risk and related ethical decision making must extend beyond procedural risk analyses alone.

A third concern relates to the public opinion of ABA services for autistic persons. As recently noted in an all-member message sent by the largest behavior analytic membership organization in the world, the relationship between ABA and “autism service interventions is increasingly under scrutiny, both within the field and in the public eye” (*Association for Behavior Analysis International* [ABAI], 2022). Some have stated concerns regarding a lack of mutually unifying best practice standards and related accountability measures (Frechter et al., 2022). In July 2021, an independent group ratified a resolution against ABA stating that it is abusive (Hyten, 2021). Expressed experiences are wide-ranging, from uncompassionate practitioner rapport to inappropriate goal selection and treatment implementation (Gibson & Douglas, 2018; Leaf et al., 2022a, b; Shkedy et al., 2021; Wilkenfeld & McCarthy, 2020). To address this issue, increased attention has been paid toward certification and accreditation requirements (e.g., BHCOE, 2022; Autism Commission on Quality, 2022; BACB, 2020).

Although enhanced regulations provide further client protections, most primarily focus on *procedures* and the validity of the achieved *outcomes* based on normative samples (i.e., outcomes determined by society rather than individual risks). A potentially far greater concern is whether practitioners are addressing the most socially meaningful *goal(s)* in the first place. Failure to do so often means adhering to a symptom-reduction approach alone (i.e., primarily based on standardized developmental and psychoeducational “excesses/deficits” with no individualization) likely further influencing increased public scrutiny regarding the field of ABA. As one example, an individualized ABA service goal to “decrease head-striking to 0 occurrences and increase the rate of requests over four weeks and across three settings” is not a complete goal because it does not include any details related to “. . . actively working to *maximize desired outcomes* and *minimize risk*” (emphasis added; BACB, 2020, p. 5). However, there currently exists no agreed-upon standards in ABA to categorize risks specifically with respect to maximizing desired service outcomes. This distinction is especially important given related implications for treatment dosage recommendations (BACB, 2019). For example, consider established practice guidelines regarding “comprehensive” (defined as treating multiple affected developmental domains as well as problem behavior) versus “focused” (i.e., treating a limited number of behavioral targets) ABA services. Some practitioners could interpret “comprehensive” and “focused” primarily with respect to narrowing the chronological-to-functional gap (Klintwall et al., 2015) but doing so is only useful if it decreases individualized risk(s) to an improved QoL, now and in the future. As such, ABA practitioners would benefit from a risk-driven framework to help determine individualized treatment dosage recommendations.

In summary, practitioners are not ethically obligated to minimize risk, but instead to “minimize risk” to “desired outcomes” (BACB, 2020, p. 5). Some practitioners may erroneously approach SMCC primarily focusing on *decreasing* risk, but an individualized ABA service goal and related desired outcomes may instead require *increasing* risk. For example, effectively addressing community-based safety skills involves increased risk exposure to improve risk-mitigation skills. Likewise, someone who has experienced trauma may want to expand their circle of support which could involve needing to learn how to take *more* risks. For example, activities like yoga and exercise have shown improvements in quality of life particularly for those who have experienced trauma (Atezaz Saeed et al., 2019; Gothe et al., 2019; Pascoe et al., 2020) and for some, successfully accessing these benefits may necessitate increasing risk (e.g., introducing risks associated with initiating a novel exercise routine). With this, the ABA community-at-large may need to expand their scope of the concept of risk to adhere to a more comprehensive risk-driven approach to SMCC, in turn helping to ensure that all involved stakeholders^4^ are putting “clients’ interests first. . .” (BACB, 2020, p. 5).

The purpose of the current article is to present two separate, yet interrelated behavior analytic conceptual analyses to (1) revisit social validity and propose features of socially meaningful case conceptualization, and (2) introduce a corresponding structured risk-driven approach to ABA service delivery. The primary goal is to equip all ABA clients and stakeholders with a readily accessible resource for maximizing individualized desired outcomes and minimizing risk (BACB, 2020). Potential contributions and limitations are discussed, including implications for future related research and practice.

## Defining Socially Meaningful Case Conceptualization (SMCC)

In the seminal article *Social Validity: The Case for Subjective Measurement or How Applied Behavior Analysis is Finding its Heart*, Wolf (1978) first described social validity as follows:

1. The social significance of the *goals*. Are the specific behavioral goals really what society wants?
2. The social appropriateness of the *procedures*. Do the ends justify the means? That is, do the participants, caregivers and other consumers consider the treatment procedures acceptable?
3. The social importance of the *effects*. Are consumers satisfied with the results? All the results, including any unpredicted ones? (p. 207)

For decades, ABA practitioners have relied on this definition of social validity to ensure stakeholder satisfaction across all aspects of service delivery. But the professional practice has since undergone significant developments. The defining features of a client's rights to effective treatment (Van Houten, 1988) were established 10 years after Wolf, both influencing subsequent certification standards and related practitioner ethical obligations. As just one example, all practitioners are now ethically obligated to base ABA service delivery on the foundation of a singular unwavering shared focus—to maximize desired outcomes and minimize risk (BACB, 2020). ABA service *goals* must now be established at the level of individualized risk identification, for the purposes of determining the most effective *procedures* and desired *outcomes*.

In addition, an ABA practitioner is now required to ensure active participation of all stakeholders across all aspects of service delivery (*Ethics Code* 2.09; BACB, 2020). An individual’s QoL can be assessed on domains such as relationships, social inclusion, personal development, self-determination, and so on (Parenti et al., 2019); however, SMCC involves the individual, their immediate and extended circles of support, and society-at-large. Under these conditions, practitioners are likely to face differing personal views on the relative value of proposed *goals* and *procedures*, and perhaps even disagreement on that which defines an improved QoL (making it nearly impossible to define desired *outcomes*). As such, practitioners would benefit from a more structured approach to ensuring a shared understanding for the needs of a given case, metrics which indicate QoL, and potential individualized risks towards accessing an improved QoL to best determine which individualized service goals and procedures are most likely to produce desired outcomes. Practitioners must also be prepared to engage in this collaborative discussion across *all* aspects of ABA service delivery, from the point of initial intake through service transition/discharge.

As one example of where current resources fall short to support SMCC, Leaf et al. (2022a, b) initiated a discussion on the appropriateness of intervening on autistic persons stereotypic behavior (a defining diagnostic feature of ASD; American Psychiatric Association, 2022). Following a description of the empirical research supporting ABA interventions for stereotypy, the authors sought to extend the dialogue on the value of risk-benefit analyses stating, “separately from the question of effectiveness is the question of whether intervening on stereotypic behavior is a good idea” (Leaf et al., 2022a, b, p. 2). In doing so, Leaf et al. further emphasized the need for practitioners to extend the concept of risk beyond *procedural* analyses alone—taking into consideration the rationale for individualized *goal* selection. The article concludes with recommendations for improved practitioner adherence to the core principles of the *Ethics Code for Behavior Analysts* (i.e., benefit others, treat others with compassion, dignity and respect, behave with integrity, and ensure competence; BACB, 2020). The authors should be commended for initiating this crucial discussion and highlighting the need for navigating potential stakeholder disagreement by listening, discussing, and collaborating to “come to agreement on goals [and] . . . work to obtain agreement of all stakeholders as to why the learning objectives will result in *quality and socially valid outcomes*. . .” (emphasis added; Leaf et al., 2022a, b, p. 13). Although Leaf et al. orient practitioners to consider all three aspects of social validity when making difficult treatment decisions, there remains limited available resources to do so.

Wolf (1978) greatly contributed to the available resources related to best practices for case conceptualization, but several potential limitations have since emerged. For example, Wolf defined social validity with respect to strengthening *goal* significance by way of establishing more robust acceptability and satisfaction measures at the level of *procedures* and *outcomes*. However, as indicated by Leaf et al. (2022a, b) we may now be facing relatively far *more* concerns regarding service *goal* selection. Wolf defined goal significance in accordance with societal-level norms without reference to (1) the individual (i.e., client/recipient of services) or (2) minimizing individualized risks to desired outcomes. Significant developments over the past several decades call into question the value associated in identifying risks with a strong focus on societal values (especially given how many views are now relatively far more diverse and often diametrically opposed). Further, the field of ABA is defined according to that which is *currently* considered to be socially meaningful for a given individual, their immediate circle of support, and the community at-large. As such practitioners are obligated to continuously revisit previously established standards and related practices to determine necessary adjustments.

Table 1 depicts a comparison between the previously established three levels of *social validity* from Wolf (1978) and the current proposed definitions for SMCC now designed to account for all involved stakeholders and potential related risks within and across all three levels. The primary difference between Wolf and the current proposed definitions (seen in the left and right columns) is the shift to a common understanding of risk at all three levels of social validity. For example, instead of “Are the specific behavioral goals really what *society* wants?” (emphasis added; Wolf, 1978) the current definitions ask, “Do all involved parties have a shared understanding for the potential risks to goal significance, appropriateness, and importance?” This requires a discussion on risk apart from the preference of a stakeholder or the norms of society in isolation in accordance with the practitioner’s ethical obligation to “in all instances . . . put clients’ interests first. . .” (BACB, 2020, p. 5).

**Table 1:**
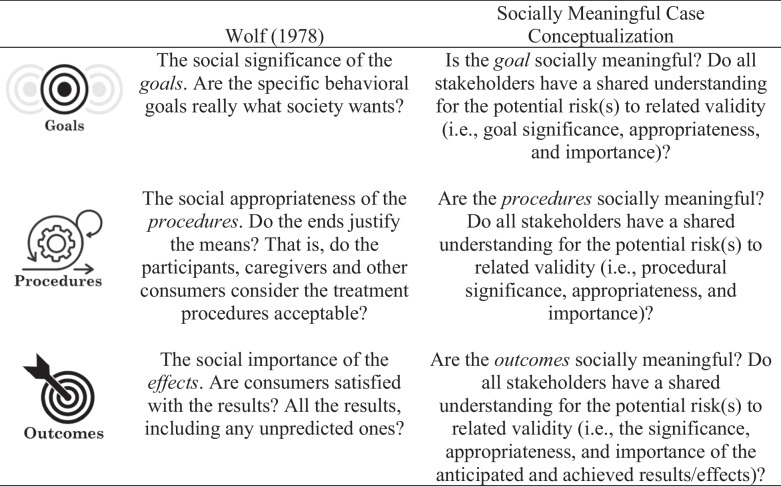
A comparison between social validity as outlined by Wolf (1978) and socially meaningful case conceptualization

It is also important to note that recent enhanced regulatory requirements (e.g., certification, accreditation, and related practice standards) have served to further increase ABA stakeholder protections (e.g., BHCOE, 2022; Autism Commission on Quality, 2022; CASP, 2020), but these improvements have focused more on the *procedural* aspect of social validity. There is less attention paid to recemmended service *goals*, and no guidance for ensuring minimized risk across all aspects of SMCC (i.e., goals, procedures and outcomes). For example, the 2022 Full Accredidation Standards established by the BHCOE includes 11 primary sections and 107 subsections (total word count: 2,988) for which there are three total references to “goal(s)” and one reference to “risk.” Although the 2020 CASP Practice Guidelines include relatively far more related references, service goal requirements are addressed one time in the 14-topic section on Critical Features of a Treatment Plan for Service Authorization and all risk-related references are specific to traditional risk/crisis management practices.

In summary, approaching the ABA case conceptualization process based on defining features of social validity that were established years before current established practice standards may set the occasion for unnecessary service delivery challenges. Expanding on the definition of social validity identified in Wolf (1978) with respect to what is “socially meaningful” (see Table 1) allows practitioners to extend the case conceptualization process beyond procedures and outcomes alone. Consumer acceptability and satisfaction measures remain paramount to quality programming, but *socially meaningful* case conceptualization will expand consideration to all three levels of social validity.

## An Introduction to The Risk-Driven Approach (TheRDA)

Understanding and taking action to address risks has become a common practice in various professional fields. The health-care field has developed Enterprise Risk Management, which defines risk as “the clinical and administrative systems, processes, and reports employed to detect, monitor, assess, mitigate, and prevent risks” (NEJM Catalyst, 2018). In a special issue on risk in social work, Whittaker and Taylor (2017) discuss risk as the “seriousness of . . . the potential harm as well as its likelihood.” They further describe the need to identify “risky situations” and the preventive actions needed to reduce the potential for harm. The Environmental Protection Agency describes risk with respect to an estimation of “the nature and probability of adverse health effects in humans . . . now or in the future” (U.S. Environmental Protection Agency, 2022). The underlying principles in these definitions pertain to the characterizing and predicting the impact of common risks within the field. Risks are certain to differ for each field given that different services entail distinctive operations.

Consider a young adult who is seeking employment, yet transportation has been identified as a potential risk to achieving this goal. Under these circumstances the individual’s immediate circle of support must collaboratively address several questions, including but not limited to, what are the potential risks involved with this individual learning to drive? Do the potential QoL improvements associated with obtaining a driver’s license outweigh the identified potential risks, and if so, what are the most effective individualized teaching procedures? Finally, how will the desired outcomes ultimately be measured (e.g., successfully obtaining a state issued license to operate a motor vehicle)? For this example, adhering to the defining features of SMCC did not equate to establishing assessment and treatment procedures aimed at the most significant QoL marker (obtaining employment). Instead, the process resulted in identifying the necessary prerequisite skills and establishing a mutually agreed upon plan for putting “clients’ interests first by maximizing desired outcomes and minimizing risk” (BACB, 2020, p. 5). This process is best described as a risk-driven approach to ABA (hereafter “TheRDA”).

### Purpose of TheRDA

TheRDA serves as the foundation for ABA services by providing a structure for determining and monitoring ABA service delivery (regardless of age, ethnicity, gender, diagnosis, socioeconomic status, severity, or service setting). TheRDA applies across the client and all stakeholders to help determine the propensity for achieving optimal ABA service outcomes. From the outset of services, a practitioner describes TheRDA as an approach to ABA-service delivery. Doing so establishes a shared understanding for the assessment process and how it relates to service recommendations. It ensures mutually agreed upon service delivery goals and clarifies expectations for service provision. TheRDA supports communication throughout the course of service delivery to guide stakeholder discussions on continued service provision. It is meant to address questions like, “Would an individual benefit from ABA?” “If someone would benefit from ABA, is it possible to deliver services?” “If services are not producing optimal outcomes, what needs to be adjusted?”

**Fig. 1.**
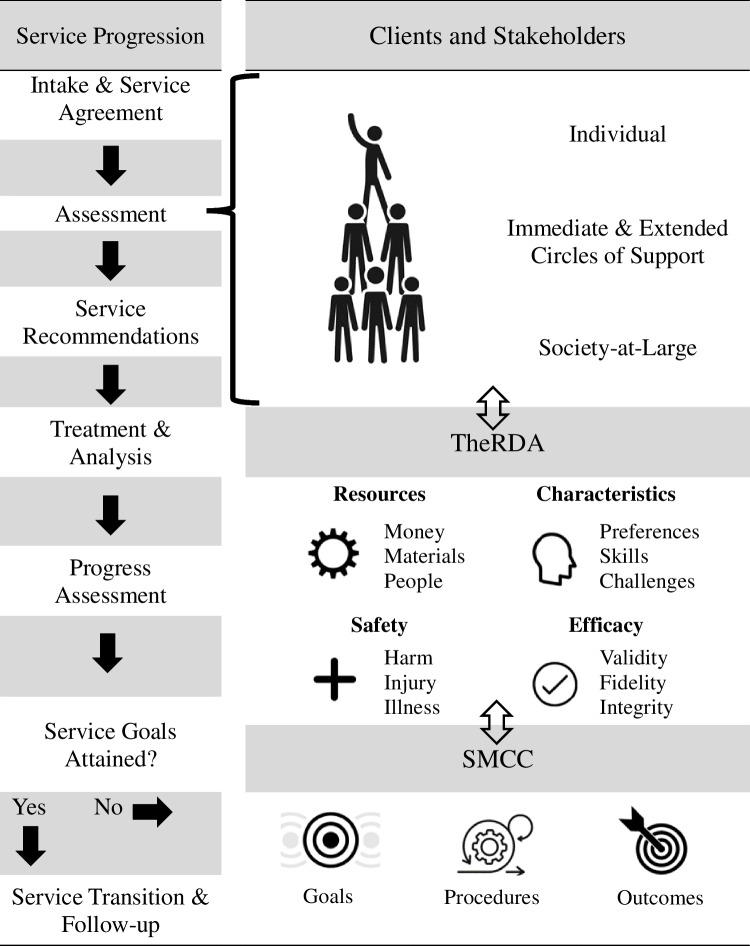
An overview of the alignment between TheRDA and the progression of ABA services

Figure 1 depicts the primary phases of TheRDA in accordance with the traditional progression of ABA services for a given individual (i.e., from the point of referral and initial intake through service transition/discharge). Across all aspects of service delivery, TheRDA process involves collaboratively determining potential risks to accessing an improved QoL (i.e., desired ABA service outcomes), and when identified, when a given risk can be (1) resolved or (2) mitigated. If the desired *outcomes* require environmental changes and adjustments (i.e., *procedural* arrangements) that necessitate substantial behavior change, ABA service recommendations must be designed in accordance with *maximizing desired outcomes* by *minimizing risk* (BACB, 2020). As an alternative, if an immediate solution is identified (i.e., an environmental change or adjustment that requires minimal corresponding behavior change) that risk does not require the development of corresponding mitigation strategies, influencing the overall ABA treatment dosage recommendations.

## TheRDA Framework

TheRDA Framework (Table 2) serves as a multifunctional tool to guide collaborative communication between all stakeholders. This tool is directly reviewed with the client and stakeholders at the point of initial intake and throughout all service delivery phases (i.e., on-going assessment, treatment, transition and/or discharge; see Fig. 1). TheRDA Framework provides a structured approach to SMCC by necessitating equal reliance on four interrelated Risk Domains: (1) Safety; (2) Resources; (3) Characteristics; and (4) Efficacy. The following sections will first include a description of TheRDA Framework followed by illustrative examples of its use.

**Table 2:**
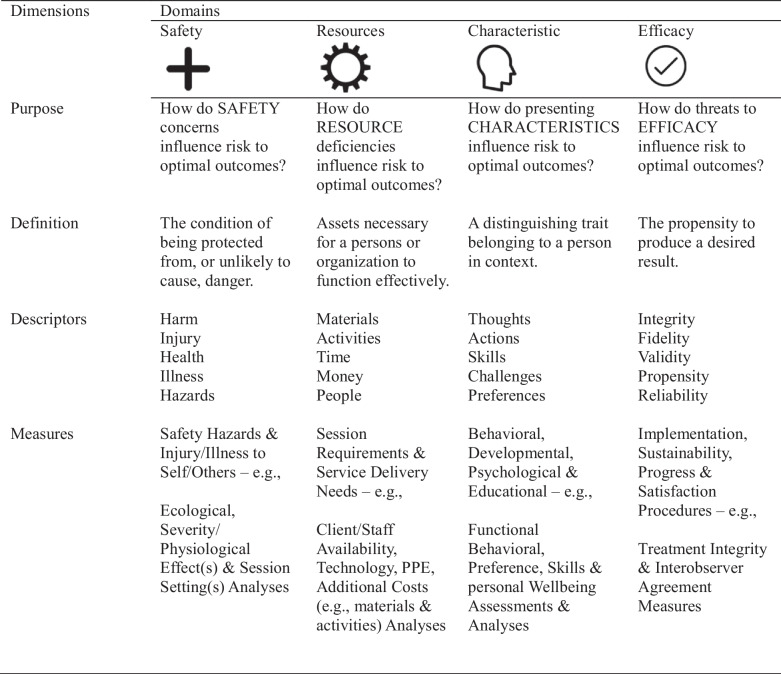
TheRDA © Framework

TheRDA Framework includes four domains (safety, resources, characteristics, and efficacy) and four dimensions (purpose, definition, descriptors, and measures)

### Risk Domains, Subdomains, and Levels

Each RDA domain provides a category for specific individually defined areas of risk (i.e., problems) related to the propensity for effective and efficient ABA-based service delivery (Table 2). The Safety domain sets the occasion for practitioners to consider how service delivery will influence the potential for injury, hospitalization, incarceration, illness, and/or death. The Resource domain helps determine what is needed to effectively deliver services. Information on Characteristics will define aspects of the consumer (such as QoL skills; e.g., communication, self-help) along with stakeholders (e.g., ability to maintain treatment procedures). Finally, the Efficacy domain ensures the practitioners will evaluate the likelihood for successful service delivery under the context of the current social environment (e.g., caregivers’ confidence in the treatment plan, understanding of aba principles).

Each Risk Domain is detailed according to four subdomains: Purpose, Definition, Descriptors, and Measures. In describing these domains and subdomains to a designated caregiver, the practitioner explains each domain-specific purpose and definition and how the additional subdomains relate: Descriptors identify terms related to each domain, and Measures describe how a particular risk is measured per each domain (e.g., an ecological assessment for resources, or a QoL assessment for characteristics).

Table 3 depicts TheRDA Risk Levels: High, Moderate, and Low. Adhering to these Levels across all RDA Domains further supports more effective stakeholder communication. In particular, practitioners are required to rely on myriad clinical assessment and programming processes, and although there exists a variety of commonly employed ABA-based *tools* and *procedures* (e.g., Verbal Behavior Milestones Assessment and Placement Program, Essential for Living, Reinforcer Assessment for Individuals with Severe Disabilities, Functional Analysis Screening Tool, World Health Organization Quality of Life Assessment, The PEAK Relational Training System), there are no established comprehensive methods for evaluating all outcomes in a consistent manner. There is need for a structured approach to summarizing all employed measures according to High, Moderate, and/or Low levels of risk throughout all phases of ABA service assessment and delivery.

**Table 3:** TheRDA © Risk Levels

	Risk Levels		
	High	Moderate	Low
Definition	Significant risk concerns Risk(s)/risk indicators cannot be completely ameliorated; reliable risk mitigation strategies are extremely impeded.	Some risk concerns Risk(s)/risk indicators can likely be ameliorated; reliable risk mitigation strategies somewhat impeded.	Minimal risk concerns Risk(s)/risk indicators can likely be ameliorated; reliable risk mitigation strategies can be implemented with no barriers

TheRDA definitions for high, moderate, and low levels of risk

In summary, adhering to TheRDA Framework and related Risk Levels provides a structured approach to putting “clients’ interests first . . . to maximize desired outcomes and minimize risk” (BACB, 2020). Doing so helps to ensure a shared understanding among all stakeholders for risks to skills such as self-determination, self-advocacy, and independence—all indicators of improved QoL, regardless of symptomology. Individualized Risk Levels are initially determined based on a comprehensive evaluation of the employed assessment sources that when taken together serve to generate data-based recommendations for individualized service intensity and delivery format. The practitioner continues to rely on the Levels throughout all phases of service delivery (e.g., continuous review and analysis of on-going treatment outcomes, including regularly scheduled formal progress report reviews). The Risk-Levels are designed to protect service eligibility regardless of the identified degree of risk exposure and/or propensity for demonstrating appropriate risk mitigation skills—each identified risk exposure is directly influenced by the degree of a given individual’s exposure to other related areas of risk and the propensity for amelioration or the development of skills associated with risk mitigation (e.g., identified areas of Low risk in certain areas, but High in others, likely indicates the need for services that align with an overall identified Moderate level of risk).

### Using TheRDA Framework

The development of TheRDA was influenced by several identified potential gaps in the available ABA-based practitioner resources for adhering to a SMCC process, including but not limited to, best practice standards for (1) treatment dosage recommendations; (2) stakeholder involvement and communication; and (3) the appropriate progression of services (e.g., process for determining a potential increase/decrease in the service delivery frequency and/or intensity). To provide some example context for the applicability of TheRDA, two scenarios are introduced here with a description of how TheRDA would affect the situation.

- ***Scenario #1: Treatment Dosage Recommendations***
  Two caregivers recently completed a comprehensive intake and assessment process for their recently diagnosed child to potentially receive in-home ABA services. When the practitioner presents their report and related service recommendations, the caregivers ask how the assessment results (from what felt like countless surveys, questionnaires, structured observations, and so on) will inform the recommended service hours. How does TheRDA inform this aspect of the practitioner’s responsibility?
- ***Scenario #1 with TheRDA: Treatment Dosage Recommendations***
  Relying on a single marker/metric (i.e., risk) helps to establish consensus among all involved parties regarding treatment dosage recommendations. For this scenario, TheRDA provides a structured approach to summarizing the results of assessment and intervention tools according to High, Moderate, and/or Low Risk within and across four clearly defined Risk Domains. The practitioner adheres to TheRDA Framework to explain how relative Risk Levels are used to inform service delivery recommendations in accordance with individualized needs towards an improved QoL. The recommendations account for the anticipated frequency and duration of ABA service hours (i.e., relatively low, moderate, or high intensity in accordance with related established best practice standards). TheRDA also increases the likelihood for all involved parties to avoid relying on a symptom-reduction approach given the clear emphasize on minimizing environmental risks related to Safety, Resources, Characteristics, and Efficacy. The purpose of an ABA assessment is not to reconfirm the diagnostic symptoms. Doing so places the locus of control within the child that can readily influence a practitioner to erroneously navigate the SMCC process by focusing on how to change the individual to achieve desired outcomes, versus modifying the environment to ensure improved QoL.
- ***Scenario #2: Stakeholder Communication***
  An individual has been demonstrating increased sexual activity, including isolating themselves for many hours and masturbating. The individual is refusing to participate in other activities and will often avoid hygienic practices. The legally responsible caregiver is vehemently opposed to supporting any type of potentially sexual activity. Instead, the caregiver offers other goals based on what they personally perceive to be appropriate markers for an improved QoL such as cleaning, outdoor activities, and academic tasks. When the ABA practitioner attempts to address the increasing pattern of isolation due to masturbation, the caregivers restate their opposition to addressing that topic. How does TheRDA support a practitioner in this scenario?
- ***Scenario #2 with TheRDA: Stakeholder Communication***
  Relying on TheRDA Framework helps to establish consensus regarding the defining features of SMCC. For this scenario, doing so (1) necessitates an increased focus on ensuring that the primary client (recipient of services) has a critical role in the SMCC process; (2) ensures that all involved parties are familiar with an ABA providers’ ethical obligation to put “*clients’ interests first* by actively working to . . . *minimize risk*” (emphasis added; BACB, 2020, p. 5); and (3) orients the caregivers to how their personal preferences and/or values (i.e. Characteristics) may pose an increased risk towards achieving desired outcomes. Adhering to TheRDA allows the practitioner to respond to the caregivers by saying something like, “Ok, those are great potential service goals! My job is to ensure that we are minimizing all potential risks to desired outcomes. If we want to address these other goals, we will need a way to discuss the recent increase in masturbation/isolation and related risks to an improved QoL.” From there, the practitioner can now more readily discuss (1) potential Safety Domain risks (e.g., viral infection due to avoiding hygienic practices); (2) the purpose of the Characteristics Domain (e.g., to assess and discuss both client and parent preferences and related values); and (3) risk indicators regarding putting a clients’ interests first (BACB, 2020). Although this scenario may not result in mutual agreement (given caregiver versus client preferences) adhering to TheRDA provides a structured approach to establishing consensus regarding the scope and nature of services, and helping all involved parties to rely on a foundation of best practice standards and related ethical obligations.

A primary contribution of TheRDA is the protection it affords against potentially relying too heavily on the interests of a single stakeholder. A given stakeholder can hold a strong influence on the priorities of treatment based on their own personal preference, sometimes with limited understanding for the individual’s personal values. On the other side, some individuals may not understand how their personal goals and related values may or may not align with those in their immediate and extended circles of support. TheRDA provides practitioners with a structured way to navigate these and related potentially challenging conversations among all involved stakeholders through the language of risk.

Adhering to TheRDA Framework throughout service delivery also helps practitioners to further protect against limiting their scope of analysis. For example, given that practitioner training heavily emphasizes the importance of conducting functional contextual analyses for the purposes of identifying equivalent replacement behaviors,^5^ many practitioners may erroneously be overly focused on the Characteristics Domain in isolation. The other Domains may only be considered based on the results of those domain-specific tools and procedures (e.g., FBA and skills assessments alone). Some practitioners may also be more likely to focus the case conceptualization process solely at the level of the individual client, only taking the other stakeholders into consideration for the purposes of caregiver training (i.e., per related payor requirements, e.g., CASP, 2020). Doing so not only more closely resembles adhering to a symptom-reduction approach, but it also places all stakeholders at an increased risk of failing to achieve an improved QoL. All four Risk Domains are interrelated and each influences all stakeholders.

TheRDA contributes to current related literature in ABA by giving practitioners a structured approach to summarizing the outcomes of their assessment, communicating the priorities of the case objectively to stakeholders, clearly outlining the projection of services, and maintaining consistent underlying goals throughout treatment. Previous literature on risk in ABA is not replaced by TheRDA but can fit within the framework. For example, a practitioners might use the risk/benefit analysis described in Deochand et al. (2020) to determine whether an FA is appropriate in the relevant context (assessing clinical experience, environment, support staff, and behavior intensity). The information gathered in this analysis can be used to inform other aspects of the case. If the risk/benefit analysis shows a lack of support staff to conduct the FA, this would be categorized as a risk in the Resource domain of TheRDA—adding to the overall risk of effective treatment outcomes. As the practitioners continues to add information to TheRDA, it will allow the practitioners to better predict the outcomes of the case and benefit all involved parties.

## General Discussion

The purpose of this article was two-fold: (1) revisit social validity and propose features of socially meaningful case conceptualization; and (2) introduce a corresponding structured risk-driven approach to ABA service delivery. TheRDA is meant to equip all stakeholders with readily accessible practice-related supports to (1) adhere to a clearly defined approach to SMCC (i.e., clinically programming with respect to maximizing desired outcomes and minimizing risk according to individualized goals, procedures and outcomes across all involved stakeholders); (2) assess and address individualized risk(s) to an improved QoL across all phases of ABA service delivery; (3) categorize the outcomes of a wide range of assessment tools according to high, moderate, and/or low risk; and (4) improve overall communication among all stakeholders. Although the current article marks several potential contributions to the available literature and related practitioner resources, there are some potential limitations worth noting.

The primary purpose of the current article was not to provide an empirical evaluation but instead a conceptual approach based on behavior analytic principles, the BACB *Ethics Code*, contributions from subject matter experts, and a reliance on scientific attitudes (e.g., parsimony, determinism). Although there are well-established approaches for non-data-based behavior analytic publications (e.g., Baer et al., 1968; Van Houten et al., 1988; Wolf, 1978), corresponding experimental analyses on the generalizability of TheRDA are warranted.

A formal research investigation would also serve to address another potential limitation related to the background and development of TheRDA. The primary content development contributors represent various areas of expertise in the field of ABA and contributed through structure and informal discussions. Aspects of TheRDA (such as risk levels) received pilot testing to help identify a client’s specific risks to their QoL. Although this input and testing is based on previous discussion and empirical studies, future researchers are especially encouraged to conduct formal empirical evaluations based on active and on-going all stakeholder input.

There are also several implications for future related practice worth noting. For example, TheRDA’s more expanded scope of the concept of risk may support improved treatment dosage recommendations. Identified high, moderate, and/or low levels of risk within and across each established domain can be relied on for the purposes of determining appropriate session frequency and duration, allowing for potentially more efficient decision making with respect to initial and on-going service delivery. There are also implications related to protecting against a narrowly focused case conceptualization process. For example, determining the most appropriate individualized goal in isolation and/or solely based on societal-level values does not equate to that which defines *socially meaningful*. Finally, there are several implications regarding public opposition to ABA. For example, adhering to TheRDA for SMCC protects an individual’s right to effective treatment by always putting “clients’ interests *first* . . .” (emphasis added; BACB, 2020, p. 5), which is in direct alignment with other approaches to providing meaningful supports, such as self-determination and person-centered planning (Parenti et al., 2019).

In closing, TheRDA is as a behavior analytic conceptual framework designed to protect against a symptom-reduction lens for socially meaningful case conceptualization. This approach further elevates individualized ABA service recommendations by requiring practitioners to move beyond identified deficits alone, necessitating equally robust analyses across ABA service *goals* and *procedures* to *maximize desired outcomes and minimize risk*. The implications for future related research and practice are robust, providing direct support towards further protecting clients’ rights to effective treatment, and in turn, the integrity of the field of ABA at-large.

## Data Availability

Not applicable.

## Footnotes

1 The defining features of QoL include, but are not limited to, social inclusion, personal development, self-determination (Parenti et al., 2019).

2 *Ethics Code for Behavior Analysts* 2.01 “Providing Effective Treatment: Behavior analysts prioritize clients’ rights and needs in service delivery. They provide services that are conceptually consistent with behavioral principles, based on scientific evidence, and designed to maximize desired outcomes. . .” (BACB, 2020).

3 Refer to BACB *Ethics Codes* 2.13, 2.14, and 3.01 (2020).

4 The term “stakeholder” is used in this paper in accordance with the definition provided in the BACB *Ethics Code*. Some are beginning to use other terms (e.g., “interested party”) due to some negative implications of the term, “stakeholder.” Future discussions are recommended to provide clarity.

5 Refer to BACB *Ethics Code* 2.14 for functional contextual assessments for replacement behavior.

## References

[CR1] American Psychiatric Association. (2022). *Diagnostic and statistical manual of mental disorders* (5th ed., text rev.).

[CR2] Association for Behavior Analysis International. (2022). Contingent electric shock task force. https://www.abainternational.org/about-us/organizational-chart/task-forces.aspx

[CR3] AtezazSaeed S, Cunningham K, Bloch RM (2019). Depression and anxiety disorders: Benefits of exercise, yoga, and meditation. American Family Physician.

[CR4] Autism Commission on Quality. (2022). ACQ Applied behavior analysis accreditation program standards and guide (version 1.0). Retrieved January 15, 2023, from https://autismcommission.org/standards/

[CR5] Axelrod S, Spreat S, Berry B, Moyer L, Van Houten R, Axelrod S (1993). A decision-making model for selecting the optimal treatment procedure. Behavior analysis and treatment.

[CR6] Baer DM, Wolf MM, Risley TR (1968). Some current dimensions of applied behavior analysis. Journal of Applied Behavior Analysis.

[CR7] Behavior Analyst Certification Board. (2019). *Clarifications regarding applied behavior analysis treatment of autism spectrum disorder: Practice guidelines for healthcare funders and managers* (2^nd^ ed.). Retrieved January 15, 2023, from https://www.bacb.com/wp-content/uploads/2020/05/Clarifications_ASD_Practice_Guidelines_2nd_ed.pdf

[CR8] Behavior Analyst Certification Board. (2020). Ethics code for behavior analysts. Retrieved January 15, 2023, from https://bacb.com/wp-content/ethics-code-for-behavior-analysts/

[CR9] Behavior Analyst Certification Board. (n.d.). BACB certificant data. Retrieved January 15, 2023, from https://www.bacb.com/BACB-certificant-data

[CR10] Behavioral Health Center of Excellence. (2022). BHCOE standard 201: Standards of excellence for applied behavior analysis services. Retrieved January 15, 2023, from https://www.bhcoe.org/standard/bhcoe-standard-201-standards-guidelines-for-effective-applied-behavior-analysis-organizations/

[CR11] Clancy, K., & Plavnick, J. (2020). *Risk assessment and mitigation strategies for applied behavior analysis: treatment of children with autism during a pandemic*. Michigan Taskforce on ABA Treatment during the Pandemic. Retrieved January 15, 2023, from https://ddi.wayne.edu/covid19/aba_risk_management_document_revised_and_disseminated_july_2020.pdf

[CR12] Colombo RA, Taylor R, Hammond JL (2020). State of current training for severe problem behavior: A survey. Behavior Analysis in Practice Journal.

[CR13] Colombo RA, Wallace M, Taylor R (2020). An essential service decision model for ABA provider during crisis. Behavior Analysis in Practice Journal.

[CR14] Council of Autism Service Providers. (2020). Applied behavior analysis treatment of autism spectrum disorder: Practice guidelines for healthcare funders and managers. Retrieved January 15, 2023, from https://casproviders.org/wp-content/uploads/2020/03/ABA-ASD-Practice-Guidelines.pdf

[CR15] Cox DJ, Brodhead MT (2021). A proof of concept analysis of decision-making with time-series data. The Psychological Record.

[CR16] Cox DJ, Plavnick JB, Brodhead MT (2020). A proposed process for risk mitigation during the COVID-19 pandemic. Behavior Analysis in Practice Journal.

[CR17] Deochand N, Eldridge RR, Peterson SM (2020). Toward the development of a functional analysis risk assessment decision tool. Behavior Analysis in Practice Journal.

[CR18] Frechter Y, Demirsoy I, Cameron MJ, Wirtjes P (2022). Toward a value-based care model for children with autism spectrum disorder. OPAST.

[CR19] Gibson, M. F., & Douglas, P. (2018). Disturbing behaviors: Ole Ivar Lovaas and the queer history of autism science. *Catalyst: Feminism, Theory, Technoscience, 4*, 1–28. 10.28968/cftt.v4i2.29579

[CR20] Gothe NP, Khan I, Hayes J, Erlenbach E, Damoiseaux JS (2019). Yoga effects on brain health: A systematic review of the current literature. Brain Plasticity.

[CR21] Hajiaghamohseni Z, Sweeney J, Anderson MC, Duarte S, Evanko C (2022). Continuum of care screener: A risk mitigation tool to guide decision making when environmental factors affect service delivery. Behavior Analysis in Practice Journal.

[CR22] Hanley GP (2012). Functional assessment of problem behavior: Dispelling myths, overcoming implementation obstacles, and developing new lore. Behavior Analysis in Practice.

[CR23] Hyten, A. (2021). Resolution opposing applied behavior analysis. Advocacy Monitor. Retrieved January 15, 2023, from https://advocacymonitor.com/ncil-resolution/resolution-opposing-applied-behavioral-analysis-aba/?fbclid=IwAR0S-VB--9EjcXvMbXPqKl7vhq1C980OWkK5yM-LNKJPxKJ-ElypKFswyx0

[CR24] Klintwall L, Eldevik S, Eikeseth S (2015). Narrowing the gap: Effects of intervention on developmental trajectories in autism. Autism.

[CR25] Leaf JB, Cihon JH, Javed A, Klick S, Ferguson JL, Milne C, Creem A, Arthur S, Saunders MS, Olive ML, Ross RK, Leaf R, McEachin J (2022). A call for discussion on stereotypic behavior. European Journal of Behavior Analysis.

[CR26] Leaf JB, Cihon JH, Leaf R, McEachin J, Liu N, Russell N, Unumb L, Shapiro S, Khosrowshahi D (2022). Concerns about ABA-based intervention: An evaluation and recommendations. Journal of Autism & Developmental Disorders.

[CR27] NEJM Catalyst. (2018). What is risk management in healthcare. Catalyst.nejm.org. Retrieved January 15, 2023, from https://catalyst.nejm.org/doi/full/10.1056/CAT.18.0197

[CR28] Parenti, K., Weiss, M. J., Nipe, T., & Southwick, J. (2019). Clinical corner: Defining and assessing quality of life as an outcome for adults with autism. *Science in Autism Treatment, 16*(5).

[CR29] Pascoe, M., Bailey, A. P., Craike, M., Carter, T., Patten, R., Stepto, N., & Parker, A. (2020). Physical activity and exercise in youth mental health promotion: A scoping review. *BMJ Open Sport & Exercise Medicine*, *6*. 10.1136/bmjsem-2019-000677 10.1136/bmjsem-2019-000677 PMC7010991 32095272

[CR30] Plavnick J. B., Clancy, K., & Milberger, S. (2020). Assessing and mitigating risk for applied behavior analysis providers during a pandemic. *Developmental Disabilities Network Journal, 1*, 210–220 10.26077/d6f1-76e2

[CR31] Reed FD, Henley AJ (2015). A survey of staff training and performance management practices: The good, the bad, and the ugly. Behavior Analysis in Practice Journal.

[CR32] Rodriguez KA (2020). Maintaining treatment integrity in the face of crisis: A treatment selection model for transitioning direct ABA services to telehealth. Behavior Analysis in Practice.

[CR33] Shkedy G, Shkedy D, Sandoval-Norton AH (2021). Long-term ABA therapy is abusive: A response to Gorycki, Ruppel, and Zane. Advances in Neurodevelopmental Disorders.

[CR34] U.S. Environmental Protection Agency. (2022). Human health risk assessment. Retrieved January 15, 2023, from https://www.epa.gov/risk/human-health-risk-assessment

[CR35] Van Houten R, Axelrod S, Bailey JS, Favel JE, Foxx RM, Iwata BA, Lovaas OI (1988). The right to effective behavioral treatment. Journal of Applied Behavior Analysis.

[CR36] Vanselow NR, Bourret JC (2012). Online interactive tutorials for creating graphs with excel 2007 or 2010. Behavior Analysis in Practice.

[CR37] Wilkenfeld DD, McCarthy AM (2020). Ethical concerns with applied behavior analysis for autism spectrum “disorder”. Kennedy Institute of Ethics Journal.

[CR38] Whittaker A, Taylor B (2017). Editorial: Understanding risk in social work. Journal of Social Work Practice.

[CR39] Wolf MM (1978). Social validity: The case for subjective measurement or how applied behavior analysis is finding its heart. Journal of Applied Behavior Analysis.

